# The role transition of radiotherapy for the treatment of liver cancer in the COVID-19 era

**DOI:** 10.3389/fonc.2022.976143

**Published:** 2022-09-14

**Authors:** Zheng Li, Yue Hu, Ming Zeng, Qinyong Hu, Fei Ye, Ruifeng Liu, Hongyi Cai, Qiang Li, Xiaohu Wang

**Affiliations:** ^1^ Institute of Modern Physics, Chinese Academy of Sciences, Lanzhou, China; ^2^ Lanzhou Heavy Ion Hospital, Lanzhou, China; ^3^ Chengdu Women’s and Children’s Central Hospital, School of Medicine, University of Electronic Science and Technology of China, Chengdu, China; ^4^ Department of Radiation Oncology, MetroHealth Medical Center, Case Western Reserve University, Cleveland, OH, United States; ^5^ Cancer Center, Renmin Hospital of Wuhan University, Wuhan, China; ^6^ Department of Radiotherapy, Gansu Provincial Hospital, Lanzhou, China

**Keywords:** COVID-19, SARS-CoV-2, liver neoplasms, radiotherapy, artificial intelligence, telemedicine, medical resource

## Abstract

The uncontrollable COVID-19 crises in the SARS-CoV-2 high-prevalence areas have greatly disrupted the routine treatment of liver cancer and triggered a role transformation of radiotherapy for liver cancer. The weight of radiotherapy in the treatment algorithm for liver cancer has been enlarged by the COVID-19 pandemic, which is helpful for the optimal risk-benefit profile.

## Highlights

1. The weight of radiotherapy in the treatment strategies for liver cancer has been enlarged by SARS-CoV-2.
2. Carbon ion radiotherapy (CIRT) possesses unique advantages to fight against SARS-CoV-2 and liver cancer synchronously.
3. CIRT or stereotactic ablative radiotherapy (SABR) could be used as a bridge for the delayed surgery due to the COVID-19 crisis.
4. Hypofractionation regimens of radiotherapy should be adequately utilized in the applicable liver cancer patients to relieve the COVID-19 pressure on limited healthcare resources in the specific areas undergoing the uncontrollable COVID-19 crisis.

## Introduction

The COVID-19 pandemic caused by SARS-CoV-2 has been going on for more than two years, inducing unprecedented social turmoil and triggering a comprehensive transformation of medical systems on a global level (1–3). After the Delta variant of SARS-CoV-2, Omicron has caused a new epidemic peak worldwide at the beginning of 2022 (3, 4). As of 19 August 2022, SARS-CoV-2 has caused more than 591.68 million confirmed cases and 6.44 million deaths according to the reported data by WHO (4). In addition, the COVID-19 excess mortality collaborators estimated that 18.2 million people died worldwide due to the COVID-19 pandemic between Jan 1, 2020, and Dec 31, 2021 (1), which is far more than the death number reported by WHO.

Pandemics in human history, such as the 1918 H1N1 pandemic with an estimated 50 million deaths as a representative (5), have taught us it is necessary to remain vigilant against the COVID-19 pandemics and do everything we can to save as many lives as possible. Timely life-saving is the foremost principle to reduce death in the context of the unprecedented COVID-19 crisis worldwide. Therefore, various novel solutions have been proposed to fight against SARS-CoV-2. Dhawan *et al. (* 6) presented a novel idea of low-dose radiotherapy as a potential lifesaving treatment for COVID-19-induced acute respiratory distress syndrome based on substantial correlational studies. Verna *et al. (* 7) indicated that COVID-19 should become the preferred research subject in response to this pandemic. As a matter of fact, the routine treatment of liver cancer was disrupted by the COVID-19 pandemic (8, 9). The dilemma of liver cancer treatment needs to be noticed and further explored (10). Therefore, we expounded the role transition of radiotherapy in this mini review, which is a pragmatic solution for the balance between the risk of SARS-CoV-2 infection and malignant death of liver cancer during the unprecedented COVID-19 crisis.

## The dilemma of liver cancer treatment

The liver cancer patients receiving surgery are at higher risk of SARS-CoV-2 infection because of their immunocompromised state and other poor holistic conditions (11, 12). Moreover, it is associated with worse prognosis when liver cancer patients are infected with SARS-CoV-2 concomitantly (11). In fact, routine surgery is unavailable for some appropriate patients with liver cancer because of the high risk of SARS-CoV-2 infection for both surgeons and patients in the specific areas undergoing the uncontrollable COVID-19 crisis (SAUCCC) (8, 9, 13, 14). On the other hand, the malignant death of liver cancer patients has greatly increased due to giving up or postponing surgery, a compromise strategy occurring because of the COVID-19 pandemic (8, 9, 15, 16). What is the solution for the dilemma to balance the risk of SARS-CoV-2 infection and the risk of malignant death of liver cancer?

## Radiotherapy for liver cancer in the COVID-19 era

Maybe the COVID-19 era is a primetime for the single-fraction (or shortened-course) radiotherapy in treating applicative liver cancer, which has been placed great expectations (10, 17, 18). Aitken *et al. (* 19) proposed that surgery for the patients with liver cancer could be provisionally replaced by the non-invasive stereotactic ablative radiotherapy (SABR) during the unprecedented COVID-19 crisis. Moreover, SABR could offer outpatient ablative approach with minimal hospital footfall and with lower immunosuppressive risk than chemotherapy, which is significant to minimize the risk of SARS-CoV-2 infection in the SAUCCC (19). Some preliminary evidence revealed that SABR could offer comparable anticancer effectiveness compared with surgery (20–22). However, the conservative paradigm, SABR is only used as a bridge for the delayed surgery due to COVID-19 (23), should be considered as the preferred strategy until we obtain the conclusive evidence of comparison between SABR and surgery. Compared with SABR, carbon ion radiotherapy (CIRT) is a better alternative strategy to the delayed surgery for liver cancer due to its multidimensional superiorities (10, 24–26), including target conformity, immune system and normal liver tissues sparing, relative biological effectiveness (RBE), total duration of treatment, and so on.

The inherently physical and biological superiorities enable CIRT to break through the limitations of conventional radiotherapy modalities in the treatment of liver cancer, maximize the anti-cancer efficacy while minimizing hepatotoxicity and immune-toxicity (10, 25, 26). CIRT possesses unique advantages to fight against COVID-19 and liver cancer synchronously in the SAUCCC (10, 24). Cutting off transmission routes could be well realized in the process of CIRT by the environmental eradication of SARS-CoV-2 and the remote treatment with the help of ultra-modern artificial intelligence (including robot) in the future (10, 27, 28). CIRT also has the superior capacity to protect vulnerable populations (patients with liver cancer) in the SAUCCC, which could be summarized as follows (10, 24). (a) The peculiarities of precision and non-invasion enable CIRT to preserve a better overall status (including immunity) for liver cancer patients to resist SARS-CoV-2 infection. (b) The duration of hospitalization, a crucial factor associated with the risk of nosocomial cross-infection of SARS-CoV-2, could be significantly shortened by CIRT because of its fewer fractions and shorter course than conventional radiotherapy modalities. (c) Single-fraction (or shortened-course) CIRT by the outpatient ablative approach could minimize the risk of SARS-CoV-2 infection by minimizing the exposure frequency and total duration of nosocomial SARS-CoV-2 source. Therefore, single-fraction (or shortened-course) CIRT would be the optimal strategy for some specific patients with liver cancer during the COVID-19 crisis.

SARS-CoV-2 Delta and Omicron Variants have caused unprecedented waves of epidemic peak respectively in 2021 and 2022 (3, 4). A viewpoint claimed that the toll on non-COVID-19 patients will be much greater than COVID-19 deaths (16). In fact, the death of both patients with COVID-19 and without COVID-19 greatly increased when the medical resources became unprecedented shortage due to the severe COVID-19 epidemics and the sequelae of COVID-19 (1, 2, 16, 29–32). Accordingly, it is imperative to improve the utilization rate of inpatient and outpatient care in the SAUCCC. The similar predicament of liver cancer patients could be alleviated by CIRT on account of its superior turnover rate of hospitalization and the single-fraction (or few fractions) capacity of outpatient treatment (10, 24). However, currently only a few countries have the equipment and technology of CIRT, including Japan, Germany, Italy, China, Austria, and America. Therefore, photon and proton SABRs are the feasible alternative strategies to CIRT in the regions without CIRT resource (18–20, 33). In order to achieve the above advantages of CIRT, hypofractionation regimens for photon and proton radiotherapy should be adequately utilized to shorten treatment schedules for liver cancer patients in the SAUCCC when feasible and appropriate (18, 33, 34). Compared with photon, proton radiotherapy has the superiority of Bragg peak that allows for the pinpoint delivery of maximum sharp energy deposition to tumors while minimizing toxicity to the surrounding noncancerous tissues and organs. Therefore, the optimized potentiality of proton radiotherapy, such as the novel FLASH approach with pencil beam scanning (33), should be fully appreciated and exploited for liver cancer patients in the SAUCCC.

There are many treatment modalities for patients with liver cancer. The role of radiotherapy was very limited in the treatment of liver cancer before the COVID-19 pandemic (17). However, the weight of radiotherapy in the treatment algorithm has been enlarged by SARS-CoV-2 (17). Systemic chemotherapy is associated with significantly deteriorated conditions of immune-system, high risk of SARS-CoV-2 infection and worse prognosis of patients with COVID-19 concomitant (11, 12). Compared with SABR (CIRT), surgical resection emerged the limitation of higher risk of SARS-CoV-2 infection in the SAUCCC (10, 17, 28). Compared with surgery by the surgical team in the operating room, SABR (CIRT) has a unique advantage on account of its special ability to treat patients with isolation in the radiation delivery compartment, which should be fully exploited and further enlarged with the help of artificial intelligence (including robot) in the SAUCCC (10, 17, 28). As we all know, artificial intelligence has made a breakthrough for the COVID-19 prevention and control in the pandemic (27, 28, 35, 36). The risk of SARS-CoV-2 infection during the course of radiotherapy will become more controllable in the SAUCCC when radiotherapy could realize completely remote treatment with the help of ultra-modern artificial intelligence in the future (27, 28).

## Discussion

It is unclear whether Omicron BA.2, BA.4, or BA.5 will trigger a new epidemic peak. It is also unclear whether new-type and more dangerous variants of SARS-CoV-2 will appear based on the large base of global COVID-19 patients (37, 38). However, it is definitely clear that more than six million deaths at least were directly caused by COVID-19, which should not be overlooked as an inanimate number (1, 4). Each death number is an irreplaceable life with a potential broken family behind it. In addition, the sequelae of COVID-19, probably including severe acute hepatitis in children emerged in some countries (39), are continually adding to the healthcare burden, causing further shortage of the limited healthcare resources in the SAUCCC (16, 29–32, 40, 41).

As a response to the severe COVID-19 crisis in the SAUCCC, multidimensional adjustments of radiotherapy have continuously updated to deal with the negative impact on the cancer treatment during the COVID-19 pandemic (10, 17–19, 23, 24, 28, 34, 42, 43). The role of radiotherapy in the treatment of liver cancer has undergone a significant shift due to the pandemic (10, 18), which is necessary for similar dilemmas in the future. There exists the similar role transition of radiotherapy in the treatment of lung cancer and pancreatic cancer (18, 23, 24).

Although comparative clinical trials with the control group are beneficial to provide stronger and more comprehensive evidence support for clinical decision-makings, it is difficult to carry out prospective controlled clinical trials because of medical ethics and other actual or adverse factors during the COVID-19 crisis. Actually, the currently available data are necessary for emergency decision-makings due to the timeliness and feasibility limitations of clinical trials in the real COVID-19 world.

Collectively, SABR (CIRT) is feasible to offer the optimal risk-benefit results for applicable liver cancer patients undergoing delay of surgery in the SAUCCC. We believe that personalized radiotherapy strategy after sufficient optimization will be helpful to minimize the risk of cancer malignant death and SARS-CoV-2 infection synchronously. We also believe that radiotherapy will become more and more important if we can bring its unique advantages into full play by the ultramodern technology during the unprecedented COVID-19 crisis.

## Author contributions

ZL, YH, QL, and XW developed the study conception and design. QL, XW, and MZ supervised the whole study process and coordinated all the work. All authors investigated, analyzed and synthesized the supporting data/references of the key viewpoints. All authors participated in discussion of the key viewpoints in order to form the ultimate consensus. ZL and YH wrote the initial draft. MZ, QH, FY, RL, HC, QL, and XW reviewed and revised the manuscript. All authors critically reviewed and approved the final manuscript.

## Funding

This work was jointly supported by the China Postdoctoral Science Foundation (Grant No. 2019M663860), the National Natural Science Foundation of China (Grant No. 11875299), and the Medical Science and Technology Project of Sichuan Provincial Health Commission (Grant No. 21PJ135). The funders of the study had no role in the conceptualization and design, investigation and analysis of references, viewpoints discussion, or writing of the report. The corresponding author had final responsibility for the decision to submit this paper for publication.

## Conflict of Interest

The authors declare that the research was conducted in the absence of any commercial or financial relationships that could be construed as a potential conflict of interest.

## Publisher’s note

All claims expressed in this article are solely those of the authors and do not necessarily represent those of their affiliated organizations, or those of the publisher, the editors and the reviewers. Any product that may be evaluated in this article, or claim that may be made by its manufacturer, is not guaranteed or endorsed by the publisher.

## References

[1] Wang HD Paulson KR Pease SA Watson S Comfort H Zheng P. Estimating excess mortality due to the COVID-19 pandemic: a systematic analysis of COVID-19-related mortality, 2020-21. Lancet (2022) 399(10334):1513–36. doi: 10.1016/S0140-6736(21)02796-3 PMC8912932 35279232

[2] Liu J Zhang L Yan Y Zhou Y Yin P Qi J. Excess mortality in wuhan city and other parts of China during the three months of the covid-19 outbreak: findings from nationwide mortality registries. Bmj (2021) 372:n415. doi: 10.1136/bmj.n415 33627311 PMC7900645

[3] World Health Organisation. WHO time-line COVID-19 . Available at: https://www.who.int/emergencies/diseases/novel-coronavirus-2019/events-as-they-happen (Accessed August 19, 2022).

[4] World Health Organisation. WHO coronavirus disease (COVID-19) situation dashboard . Available at: https://covid19.who.int/ (Accessed August 19, 2022).

[5] Jester B Uyeki TM Jernigan DB Tumpey TM. Historical and clinical aspects of the 1918 H1N1 pandemic in the united states. Virology (2019) 527:32–7. doi: 10.1016/j.virol.2018.10.019 30453209

[6] Dhawan G Kapoor R Dhawan R Singh R Monga B Giordano J. Low dose radiation therapy as a potential life saving treatment for COVID-19-induced acute respiratory distress syndrome (ARDS). Radiother Oncol (2020) 147:212–6. doi: 10.1016/j.radonc.2020.05.002 PMC7206445 32437820

[7] Verna EC Serper M Chu J Corey K Fix OK Hoyt K. Clinical research in hepatology in the COVID-19 pandemic and post-pandemic era: Challenges and the need for innovation. Hepatology (2020) 72(5):1819–37. doi: 10.1002/hep.31491 PMC7435542 32740969

[8] Bennett S Søreide K Gholami S Pessaux P Teh C Segelov E. Strategies for the delay of surgery in the management of resectable hepatobiliary malignancies during the COVID-19 pandemic. Curr Oncol (2020) 27(5):e501–11. doi: 10.3747/co.27.6785 PMC7606047 33173390

[9] Garg PK Kaul P Choudhary D Turaga KK Singh MP Tiwari AR. Discordance of COVID-19 guidelines for patients with cancer: A systematic review. J Surg Oncol (2020) 122(4):579–93. doi: 10.1002/jso.26110 PMC7405271 32668034

[10] Li Z Li Q Wang X Li S Chen W Jin X. Carbon ion radiotherapy acts as the optimal treatment strategy for unresectable liver cancer during the coronavirus disease 2019 crisis. Front Public Health (2021) 9:767617. doi: 10.3389/fpubh.2021.767617 34957022 PMC8695803

[11] Zhang L Zhu F Xie L Wang C Wang J Chen R. Clinical characteristics of COVID-19-infected cancer patients: a retrospective case study in three hospitals within wuhan, China. Ann Oncol (2020) 31(7):894–901. doi: 10.1016/j.annonc.2020.03.296 32224151 PMC7270947

[12] Liang W Guan W Chen R Wang W Li J Xu K. Cancer patients in SARS-CoV-2 infection: a nationwide analysis in China. Lancet Oncol (2020) 21(3):335–7. doi: 10.1016/s1470-2045(20)30096-6 PMC7159000 32066541

[13] Li Z Li Q Wang X Chen W Jin X Liu X. Hyperthermia ablation combined with transarterial chemoembolization versus monotherapy for hepatocellular carcinoma: A systematic review and meta-analysis. Cancer Med (2021) 10(23):8432–50. doi: 10.1002/cam4.4350 PMC8633247 34655179

[14] Mahase E. Covid-19: Omicron is "battering" the NHS and causing "untold suffering" for patients, say doctors. Bmj (2022) 376:o45. doi: 10.1136/bmj.o45 35012961

[15] Omarini C Maur M Luppi G Narni F Luppi M Dominici M. Cancer treatment during the coronavirus disease 2019 pandemic: Do not postpone, do it! Eur J Cancer (2020) 133:29–32. doi: 10.1016/j.ejca.2020.04.034 32434108 PMC7214318

[16] Rosenbaum L. The untold toll - the pandemic's effects on patients without covid-19. N Engl J Med (2020) 382(24):2368–71. doi: 10.1056/NEJMms2009984 32302076

[17] Scorsetti M Goodman KA Seong J Loi M Huguet F Dawson LA. Hepatocellular carcinoma in the COVID-19 era: Primetime for stereotactic body radiotherapy and a lesson for the future? Oncologist (2020) 25(8):e1249–50. doi: 10.1634/theoncologist.2020-0416 PMC7307056 32525607

[18] Ng SSW Ning MS Lee P McMahon RA Siva S Chuong MD. Single-fraction stereotactic body radiation therapy: A paradigm during the coronavirus disease 2019 (COVID-19) pandemic and beyond? Adv Radiat Oncol (2020) 5(4):761–73. doi: 10.1016/j.adro.2020.06.011 PMC7406732 32775790

[19] Aitken K Good J Hawkins M Grose D Mukherjee S Harrison M. Liver stereotactic ablative radiotherapy: an effective and feasible alternative to surgery during the COVID-19 pandemic. Clin Oncol (R Coll Radiol) (2020) 32(7):477. doi: 10.1016/j.clon.2020.04.012 32387045 PMC7252179

[20] Qiu B Aili A Xue L Jiang P Wang J. Advances in radiobiology of stereotactic ablative radiotherapy. Front Oncol (2020) 10:1165. doi: 10.3389/fonc.2020.01165 32850333 PMC7426361

[21] Chang JY Senan S Paul MA Mehran RJ Louie AV Balter P. Stereotactic ablative radiotherapy versus lobectomy for operable stage I non-small-cell lung cancer: a pooled analysis of two randomised trials. Lancet Oncol (2015) 16(6):630–7. doi: 10.1016/s1470-2045(15)70168-3 PMC4489408 25981812

[22] Chang JY Mehran RJ Feng L Verma V Liao Z Welsh JW. Stereotactic ablative radiotherapy for operable stage I non-small-cell lung cancer (revised STARS): long-term results of a single-arm, prospective trial with prespecified comparison to surgery. Lancet Oncol (2021) 22(10):1448–57. doi: 10.1016/s1470-2045(21)00401-0 PMC8521627 34529930

[23] Kidane B Spicer J Kim JO Fiset PO Abdulkarim B Malthaner R. SABR-BRIDGE: Stereotactic ABlative radiotherapy before resection to AvoId delay for early-stage LunG cancer or OligomEts during the COVID-19 pandemic. Front Oncol (2020) 10:580189. doi: 10.3389/fonc.2020.580189 33072612 PMC7544973

[24] Barcellini A Vitolo V Cobianchi L Valvo F Vischioni B Bonora M. Pancreatic cancer: Does a short course of carbon ion radiotherapy worth during COVID-19 outbreak? Pancreatology (2020) 20(5):1004–5. doi: 10.1016/j.pan.2020.05.007 PMC7217079 32418757

[25] Tinganelli W Durante M. Carbon ion radiobiology. Cancers (Basel) (2020) 12(10):3022. doi: 10.3390/cancers12103022 PMC7603235 33080914

[26] Kirkby KJ Kirkby NF Burnet NG Owen H Mackay RI Crellin A. Heavy charged particle beam therapy and related new radiotherapy technologies: The clinical potential, physics and technical developments required to deliver benefit for patients with cancer. Br J Radiol (2020) 93(1116):20200247. doi: 10.1259/bjr.20200247 33021102 PMC7715999

[27] Bhaskar S Bradley S Sakhamuri S Moguilner S Chattu VK Pandya S. Designing futuristic telemedicine using artificial intelligence and robotics in the COVID-19 era. Front Public Health (2020) 8:556789. doi: 10.3389/fpubh.2020.556789 33224912 PMC7667043

[28] Martín-Noguerol T Lopez-Ortega R Ros PR Luna A. Teleworking beyond teleradiology: managing radiology departments during the COVID-19 outbreak. Eur Radiol (2021) 31(2):601–4. doi: 10.1007/s00330-020-07205-w PMC7464047 32876832

[29] Carfì A Bernabei R Landi F. Persistent symptoms in patients after acute COVID-19. Jama (2020) 324(6):603–5. doi: 10.1001/jama.2020.12603 PMC7349096 32644129

[30] Katsoularis I Fonseca-Rodríguez O Farrington P Jerndal H Lundevaller EH Sund M. Risks of deep vein thrombosis, pulmonary embolism, and bleeding after covid-19: nationwide self-controlled cases series and matched cohort study. Bmj (2022) 377:e069590. doi: 10.1136/bmj-2021-069590 35387772 PMC8984137

[31] Jamal SM Landers DB Hollenberg SM Turi ZG Glotzer TV Tancredi J. Prospective evaluation of autonomic dysfunction in post-acute sequela of COVID-19. J Am Coll Cardiol (2022) 79(23):2325–30. doi: 10.1016/j.jacc.2022.03.357 PMC8976261 35381331

[32] Lopez-Leon S Wegman-Ostrosky T Perelman C Sepulveda R Rebolledo PA Cuapio A. More than 50 long-term effects of COVID-19: a systematic review and meta-analysis. Sci Rep (2021) 11(1):16144. doi: 10.1038/s41598-021-95565-8 34373540 PMC8352980

[33] Wei S Lin H Choi JI Press RH Lazarev S Kabarriti R. FLASH radiotherapy using single-energy proton PBS transmission beams for hypofractionation liver cancer: Dose and dose rate quantification. Front Oncol (2021) 11:813063. doi: 10.3389/fonc.2021.813063 35096620 PMC8794777

[34] Chhabra AM Choi JI Hasan S Press RH Simone CB 2nd. Prioritization of proton patients in the COVID-19 pandemic: Recommendations from the new York proton center. Int J Part Ther (2020) 6(4):38–44. doi: 10.14338/ijpt-20-00022.1 32582818 PMC7302729

[35] El-Sherif DM Abouzid M Elzarif MT Ahmed AA Albakri A Alshehri MM. Telehealth and artificial intelligence insights into healthcare during the COVID-19 pandemic. Healthcare (Basel) (2022) 10(2):385. doi: 10.3390/healthcare10020385 35206998 PMC8871559

[36] Gunasekeran DV Tseng R Tham YC Wong TY. Applications of digital health for public health responses to COVID-19: a systematic scoping review of artificial intelligence, telehealth and related technologies. NPJ Digit Med (2021) 4(1):40. doi: 10.1038/s41746-021-00412-9 33637833 PMC7910557

[37] Markov PV Katzourakis A Stilianakis NI. Antigenic evolution will lead to new SARS-CoV-2 variants with unpredictable severity. Nat Rev Microbiol (2022) 20(5):251–2. doi: 10.1038/s41579-022-00722-z PMC8919145 35288685

[38] Lino A Cardoso MA Martins-Lopes P Gonçalves HMR. Omicron - the new SARS-CoV-2 challenge? Rev Med Virol (2022) 32(4):e2358. doi: 10.1002/rmv.2358 35445774 PMC9111063

[39] Brodin P Arditi M. Severe acute hepatitis in children: investigate SARS-CoV-2 superantigens. Lancet Gastroenterol Hepatol (2022) 7(7):594–5. doi: 10.1016/s2468-1253(22)00166-2 PMC9106421 35576952

[40] Huang L Li X Gu X Zhang H Ren L Guo L. Health outcomes in people 2 years after surviving hospitalisation with COVID-19: a longitudinal cohort study. Lancet Respir Med (2022) 10(9):863–76. doi: 10.1016/s2213-2600(22)00126-6 PMC9094732 35568052

[41] Levine RL. Addressing the long-term effects of COVID-19. Jama (2022) 10(9):863–76. doi: 10.1001/jama.2022.14089 35921084

[42] Tchelebi LT Haustermans K Scorsetti M Hosni A Huguet F Hawkins MA. Recommendations for the use of radiation therapy in managing patients with gastrointestinal malignancies in the era of COVID-19. Radiother Oncol (2020) 148:194–200. doi: 10.1016/j.radonc.2020.04.010 32342878 PMC7194719

[43] Piras A Venuti V D'Aviero A Cusumano D Pergolizzi S Daidone A. Covid-19 and radiotherapy: a systematic review after 2 years of pandemic. Clin Transl Imaging (2022), 1–20. doi: 10.1007/s40336-022-00513-9 PMC9308500 35910079

